# Germination Response of Four Alien Congeneric *Amaranthus* Species to Environmental Factors

**DOI:** 10.1371/journal.pone.0170297

**Published:** 2017-01-20

**Authors:** Jian-Hua Hao, Shuang-Shuang Lv, Saurav Bhattacharya, Jian-Guo Fu

**Affiliations:** 1 Changshu Institute of Technology, Suzhou, Jiangsu, China; 2 Jiangsu Entry-Exit Inspections and Quarantine Bureau, Nanjing, Jiangsu, China; Universidad Nacional Autonoma de Mexico, MEXICO

## Abstract

Seed germination is the key step for successful establishment, growth and further expansion of population especially for alien plants with annual life cycle. Traits like better adaptability and germination response were thought to be associated with plant invasion. However, there are not enough empirical studies correlating adaptation to environmental factors with germination response of alien invasive plants. In this study, we conducted congeneric comparisons of germination response to different environmental factors such as light, pH, NaCl, osmotic and soil burials among four alien amaranths that differ in invasiveness and have sympatric distribution in Jiangsu Province, China. The data were used to create three-parameter sigmoid and exponential decay models, which were fitted to cumulative germination and emergence curves. The results showed higher maximum Germination (*G*_*max*_), shorter time for 50% germination (*G*_*50*_) and the rapid slope (*G*_*rate*_) for *Amaranthus blitum* (low-invasive) and *A*. *retroflexus* (high-invasive) compare to intermediately invasive *A*. *spinosus* and *A*. *viridis* in all experimental regimes. It indicated that germination potential does not necessarily constitute a trait that can efficiently distinguish highly invasive and low invasive congeners in four *Amaranthus* species. However, it was showed that the germination performances of four amaranth species were more or less correlated with their worldwide distribution area. Therefore, the germination performance can be used as a reference indicator, but not an absolute trait for invasiveness. Our results also confirmed that superior germination performance in wide environmental conditions supplementing high seed productivity in highly invasive *A*. *retroflexus* might be one of the reasons for its prolific growth and wide distribution. These findings lay the foundation to develop more efficient weed management practice like deep burial of seeds by turning over soil and use of tillage agriculture to control these invasive weed species.

## Introduction

The effects of invasive alien species include altering ecosystem, threatening the existence of native species, reducing biodiversity, and degrading the environment. The identification of the 0.1% of harmful invasives among important plant species within a country or a region and prioritizing control efforts according to their specific threat are the challenges we have to face [1]. It is critical, despite its difficulty to determine which exotic plant species may well become invasive ones to control them in their native ecosystem [2]. As a result, unraveling what makes a species invasive and identifying what characteristics are associated with successful establishment for invasive alien plant species are the major objectives of invasion ecology and still represents an ultimate goal of invasion ecologists [3–10].

In previous studies on identification for the traits associated with invasiveness of plant species, the comparative studies among congeneric invasive and non-invasive alien species are recognized as an effective and direct approach [5], [11], [7–9], [12]. Congeneric species are referred to the species that belong to same genus. The congeneric comparison can eliminate or reduce the biasness and variation associated with phylogenetic distance and habitat affinities of the species compared and is the better approach for identifying the traits associated with successful invaders [5], [13], [14], [6], [7], [11]. If the comparative studies were conducted by controlling for life-form, introduction history, native range of origin and habitat preferences of the study species, the results will supply better reference for invasiveness.

Seed germination is the first step of plant life cycle and the germination proportion and germination timing of seeds, major life-history traits, likely to play an important role in biological invasions [14]. Successful germination within favorable time period is crucial for establishing a population and further expansion especially in annual invasive species. [15]. In previous studies, only few experimental comparisons related to germination and early establishment were reported on some invasive and non-invasive or naturalized alien [16–18], [9], [14] and also among invasive and native [12], alien and native [5], [10], and populations of invasive species in the native and introduced ranges [19–21], [15]. Such previous comparisons highlighted different performances in germination characteristics such as rapidity and extent of germination that can be used, at least partly, to separate successful and unsuccessful invaders and deemed to be the most useful traits of evaluating potentially troublesome species[16], [22], [5], [6], [9], [10]. However, other studies also showed that there was not much difference in germination characteristic between successful and unsuccessful invaders [17], [18], [12]. Therefore to identify and further characterize any potential differences in germination traits, large number of empirical multi-species comparative studies is needed among invasive and non-invasive congeners in a common environment. [7], [11], [14].

The traits that allow a species to adapt to a wider range of environmental factors seem to be favorable for a successful invasion [4]; therefore, the invasive species that can be adapted to various conditions are more likely to have better chances to be dispersed and higher chances of invasiveness. Some experimental results of reproductive traits of congeneric pairs demonstrated that rare species were less tolerant to environmental factors than their congeneric widespread species [23–25] based on a comparative analysis of 25 ecological and biological traits in 20 congeneric pairs of endemic and widespread plant species occurring in the French Mediterranean flora. The authors found that morphological and eco-physiological traits of widespread species are often more stress-tolerant than their narrow endemics congeners.

*Amaranthus* species, commonly referred to as ‘‘pigweeds,” are among the most troublesome weeds in many crop production systems. Some results of germination ecology and response to environmental factors were reported by weed scientists [26–32]. *Amaranthus* is also the genus with several naturalized weeds including the highly invasive *A*. *retroflexus* species in China [33]. There are a few alien species among the genus differing in invasiveness, so it is an ideal studying objective for congeneric comparison for alien plants. *A*. *retroflexus* was listed as a highly invasive plant, with a risk index value of 62 and the risk rank as second grade [33]. It has been listed as one of the most noxious invasive plants in China [34]. *A*. *spinosus* L. and *A*. *viridis* L. are listed as intermediately invasive plants. Their risk indices values are 59 and 52, respectively, and the risk ranks are both third grade [33]. *A*. *blitum* didn’t appear in some inventories of invasive plants in China [35], [36], but is listed as an invasive weed with wide distribution in other inventories [37]. We defined it as a low-invasive plant.

In previous studies, the data on the influence of environmental conditions on certain germination characteristics of some *Amaranthus* spp. were reported. Thomas et al. [31] and Chauhan and Johnson [32] demonstrated the seedling emergence responses of spiny amaranth (*A*. *spinosus*) and slender amaranth (*A*. *viridis*) to temperature, light, pH solution, moisture stress, salt stress and depth of emergence. The comparative experiments on effects of temperature on seed germination of redroot pigweed (*A*. *retroflexus*), Palmer amaranth (*A*. *palmeri*), and common waterhemp (*A*. *rudis*) in regards to their population in America were showed by Guo and Al-Khatib [27], and a similar report of nine amaranth in America was published by Steckel et al. [30]. Although above mentioned data on the influence of environmental conditions on certain germination characteristics of some *Amaranthus* spp. are available, little is known on their relative invasiveness.

In this study, four congeneric alien amaranths having similar distribution area, life-form and introduction history, native range of origin and habitat preferences but differing in invasiveness were selected to study their germination and emergence responses to different environmental factors such as photoperiod, pH, osmotic potential, NaCl solution, and burial depth. The objectives of this study were (1) to test the correlation of seed response with invasiveness by comparing germination response data among four amaranths and (2) to supply the empirical evidence for considering whether seed germination response to environmental factors can be included as an indicator of risk assessment protocols for plant invasion.

## Materials and Methods

### Plant material

According to the results of Records of World Weeds [38], invasive risk indexes and ranks in China [33], invasive categories in China [37], four alien amaranths were selected which are sympatric distribution naturally in Jiangsu Province, China and have similar life-form, introduction history, native range of origin and habitat preference, but differ in invasiveness. They were redroot pigweed (*A*. *retroflexus*), spiny amaranth (*A*. *spinosus*), slender amaranth (*A*. *viridis*), and livid amaranth (*A*. *blitum*). All the selected species are annual and introduced into China in the middle of the eighteenth century (Table 1).

**Table 1:** Origin, introduction history, distribution and invasive status of four *Amaranthus* species.

Species	Origin^a^[37]	First Record [37]	Distribution Province in China [37]	Records of World Weeds^b^ [38]	Global Record^c^	Global Occurence^c^	Risk Rank in China [33]	Risk Index in China [33]	Invasive Status
***A*. *retroflexus***	Am	1753	32	S = 16, P = 16, C = 2, X = 12, F = 0	31183	36070	2nd	62	High
***A*. *spinosus***	TAm	1753	30	S = 7, P = 11, C = 18, X = 21, F = 0	2108	4847	3th	59	Intermediate
***A*. *viridis***	SAm	1763	31	S = 8, P = 14, C = 9, X = 13, F = 4	4458	6891	3th	52	Intermediate
***A*. *blitum***	TAm, Mediterranean region Europe, Asia and North Africa	1753	30	S = 0, P = 5, C = 9, X = 17, F = 0	13557	16639	-	-	Low

^a^Am = America, SAm = South America, TAm = Tropical America (Global Biodiversity Information Facility, www.gbif.org, accessed on 2016-10-7),

^b^S: Serious weed; P: Principal weed; C: Common weed; X: Present as a weed (the species is present and behave as a weed, but its rank of importance is unknown; F: Flora (the species is known to be present in the flora of the county, but confirming evidence is needed that the plant behave as a weed);

^c^Global Biodiversity Information Facility, www.gbif.org, accessed on 2016-10-7.

Seeds of four amaranths were collected from road side and abandoned areas in Yancheng (34°0′36″N, 119°49′48″E) and Nanjing (31°39′37″N, 119°15′19″E), Jiangsu Province, China in July 2011. As our four experimental species commonly grows as wild population in open areas, no legal permission needed for sampling. Seeds collected from many randomly selected plants were stored at room temperature (25°C) in paper bags until used in the experiments in February and April of 2012. The 1,000-seed weights of *A*. *retroflexus*, *A*. *spinosus*, *A*. *viridis* and *A*. *blitum* were recorded as 312mg, 129mg, 388mg, and 378mg, respectively.

### Germination tests

Germination response was determined by placing fifty seeds evenly in a 90 mm dia. Petri dish containing two pieces of filter paper. Distilled water (control set and light experiment) or the appropriate treatment solution was added to the filter paper as needed. Dishes containing seeds were incubated at 30°C-14h/25°C-10h of alternating temperatures and at 12h light/12h dark (in light experiment). This alternating temperature regime was found to be optimum among several temperature conditions tested previously in four amaranths (data not shown). Daily germination counts were made for 15 days. Each seedling was removed when a visible radicle could be discerned. The germination experiments were conducted in growth chambers with three tier racks illuminated with cool white fluorescent light (40μmm^-2^s^-1^, Philips) with 70% relative humidity (RH).

#### Photoperiod treatment

The effect of light on germination was determined by incubating seeds of four Amaranths in light/dark regimes of 0h/24h, 8h/16h, 12h/12h and 16h/8h. For germination in complete darkness, dishes were wrapped in a layer of aluminum foil.

#### pH treatment

The effect of a pH buffered solution on germination was determined by incubating seeds in dishes containing solution of pH4-pH10, which were prepared as described by Burke et al. [39] or Chauhan et al. [40]. A 100 mM Potassium hydrogen phthalate buffer solution was adjusted to pH 4, pH 5, and pH 6 with 0.1N HCl or 0.1N NaOH. A 200mM KH_2_PO_4_ buffer solution was adjusted to pH 7 or 8 with 0.1N HCl or 0.1N NaOH. A 50 mM Sodium borate buffer solution was similarly adjusted to pH 9 or 10 with 0.1N HCl or 0.1N NaOH.

#### NaCl treatment

The effects of salinity on germination response were determined by placing seeds in dishes containing aqueous solution of 0, 25, 50, 100, 150, and 200mM Sodium chloride (NaCl).

#### Water potential treatments

The effect of osmotic stress on germination was determined by incubating seeds in solutions with osmotic potentials of 0, -0.2, -0.4, -0.6 and -0.8Mpa which were prepared by dissolving 0, 112.38, 172.41, 218.13, and 256.13 g of polyethylene glycol (PEG6000) in 300 ml of distilled water [41].

#### Seed burial depth treatment

The effect of seed burial depth on seedling emergence was investigated in a greenhouse. Fifty seeds of each species were covered with soil to depths of 0, 1, 2, 3, 4, 5, and 6 cm in plastic pots (15 cm in diameter). Pots were watered initially with an overhead mist sprinkler and later sub-irrigated. Plants were watered throughout the study when the soil surfaces were dried. Seedlings were considered emerged when a cotyledon was visible on the soil surface. Emerged seedlings were counted every day up to 30 days after sowing (DAS).

### Statistical analysis

Germination performance across all treatments in all four species were analysed statistically. Each treatment had four replicates in each species. Data were analyzed using the nonlinear regression model of Sigma Plot (SigmaPlot version 11.0, from Systat Software, Inc., San Jose California USA). A three-parameter sigmoid function is used for curve-fitting to the germination data of photoperiod, pH, NaCl and water potential treatments. The formulation was as following:

G=Gmax/[1+e]−(x−T50Grate) (1)

*G* is the cumulative percentage germination at time x, *Gmax* is the maximum germination (%), *T*_*50*_ is the time (d) required for 50% of maximum germination, and *G*_*rate*_ indicates the slope.

A four-parameter sigmoid model was fitted to the germination data of seedling emergence (%) at different depths. The model fitted was as following:

E=E0+Emax/[1+e]−(x−T50Erate) (2)

*E* represents cumulative emergence (%) at time x, *E*_*0*_ is the minimum emergence (%), *E*_*max*_ is the difference of maximum and minimum emergence (%), *T*_50_ is the time (d) required for 50% of maximum emergency, and *E*_*rate*_ indicates the slope.

The main effects of differences among species and treatments were analyzed using the univariate analysis of general linear model. The significant differences among different treatments and among species were analyzed by One-Way ANOVA using SPSS 16. The significant difference among different treatments × species were analyzed by Two-Way ANOVA using SPSS16. For all analyses, differences at p<0.05 was considered significant, while p<0.01 was considered highly significant. When the test results of variances homogeneity were >0.05, the Tukey HSD were selected for multiple comparisons. When the test results of variances homogeneity were <0.05, the Dunnett C were selected for multiple comparisons.

## Results

### Light treatment

**Fig 1:**
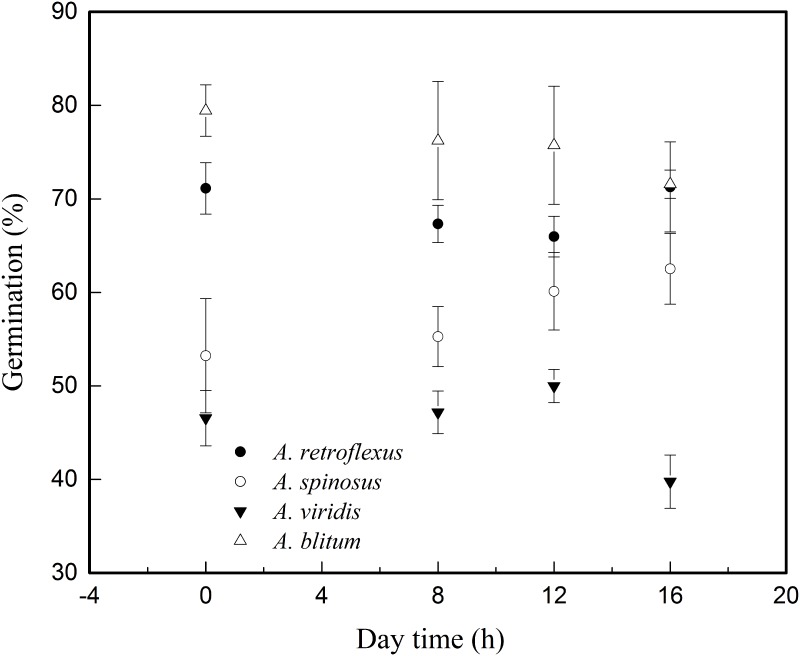
Plot showing three-parameter sigmoid model fitted data of Germination percentage (*G*_*max*_) in four *Amaranthus* species with respect to different light treatments.

Irrespective of presence or absence of light, all the four amaranths were able to germinate in our experiment (Table 2 and Fig 1); indicating that light is not an absolute requirement for their germination. The analysis of the results of main effects of general linear model showed that there was no significant difference in seed germinations (*G*_*max*_) among four photoperiods (DF = 3, F = 0.183, p>0.05), but highly significant difference noted in seed germination among four species (DF = 3, F = 62.225, p<0.01). There was no significant difference in *G*_*max*_ among photoperiod×species (DF = 9, F = 1.457, p>0.05). The analysis using multiple comparisons showed that there were no significant differences among four photoperiod treatments in all of *A*. *retroflexus* (DF = 3, F = 0.783, p>0.05), *A*. *spinosus* (DF = 3, F = 1.049, p>0.05) and *A*. *blitum* (DF = 3, F = 0.422, p>0.05). Among the four amaranth species, the only difference in light treatment was recorded in *A*. *viridis* (DF = 3, F = 3.406, p<0.05), where the seed germination response at 16/8 (day/night) light exposure were lower than that of 12/12 (day/night) light, as evident from the longer time period required for 50% germination (*T*_*50*_) and relatively high *G*_*rates*_ value showing that perhaps over-exposure to light period depressed the germination response of *A*. *viridis* seeds. Compared to other three amaranths, the time required for 50% germination (*T*_*50*_) of *A*. *viridis* were longer and the *G*_*rate*s_ values were higher (Table 2).

**Table 2:** Effect of photoperiod on the germination of four *Amaranthus* species, incubated at 30/25°C in light/dark.

Species	Parameters	Day time (h)			
		0	8	12	16
***A*. *retroflexus***	*G*_*max*_ (%)	72.90(2.74)^**a,k**^	70.40(1.98)^**a,kl**^	67.50(2.17)^**a,kl**^	72.70(4.81)^**a,k**^
	*G*_*rate*_	0.32(0.04)	0. 14(0.16)	0.16(0.08)	0.16(0.10)
	*T*_*50*_(d)	1.96(0.03)	1.78(0.25)	1.82(0.09)	1.80 (0.12)
	*R*^*2*^	0.9962	0.9937	0.9967	0.9954
***A*. *spinosus***	*G*_*max*_ (%)	54.50 (6.10)^**a,l**^	61.20 (3.21)^**a,lm**^	60.50 (4.14)^**a,lm**^	66.30 (3.79)^**a,k**^
	*G*_*rate*_	0.37 (0.02	0.22 (0.12)	0.12 (0.12)	0.20 (0.05)
	*T*_*50*_ (d)	2.22 (0.02)	1.79 (0.13)	1.80 (0.19)	1.89 (0.03)
	*R*^*2*^	0.9985	0.9711	0.9990	0.9978
***A*. *viridis***	*G*_*max*_ (%)	52.40 (2.95)^**ab,l**^	54.70 (2.29)^**ab,m**^	58.60 (1.78)^**a,m**^	46.0 (2.85)^**b,l**^
	*G*_*rate*_	0.79 (0.09)	1.47 (0.12)	1.27 (0.18)	2.23 (0.16)
	*T*_*50*_ (d)	4.51 (0.10)	6.06 (0.14)	4.15 (0.21)	8.92 (0.24)
	*R*^*2*^	0.9913	0.9920	0.9739	0.9944
***A*. *blitum***	*G*_*max*_ (%)	79.20 (2.75)^**a,k**^	78.20 (6.32)^**a,k**^	77.00 (6.31)^**a,k**^	72.50 (1.51)^**a,k**^
	*G*_*rate*_	0.34 (0.05)	0.10 (0.23)	0.08 (0.69)	0.11 (0.24)
	*T*_*50*_ (d)	2.48 (0.02)	1.78 (0.48)	1.82 (1.62)	1.77 (0.48)
	*R*^*2*^	0.9994	0.9991	0.9999	0.9970

Table showing parameter estimates [*G*_*max*_, maximum germination (%); *G*_*rate*_, slope; *T*_50_, time to reach 50% of maximum germination (days)] of seed germination.

Values represent mean and standard error (parentheses).

Significant differences in G_*max*_ were indicated by letters a-b, among photoperiod treatments within same species (comparison within row) and letters k-m, among species within same photoperiod treatment (comparison within column).

### pH treatment

**Fig 2:**
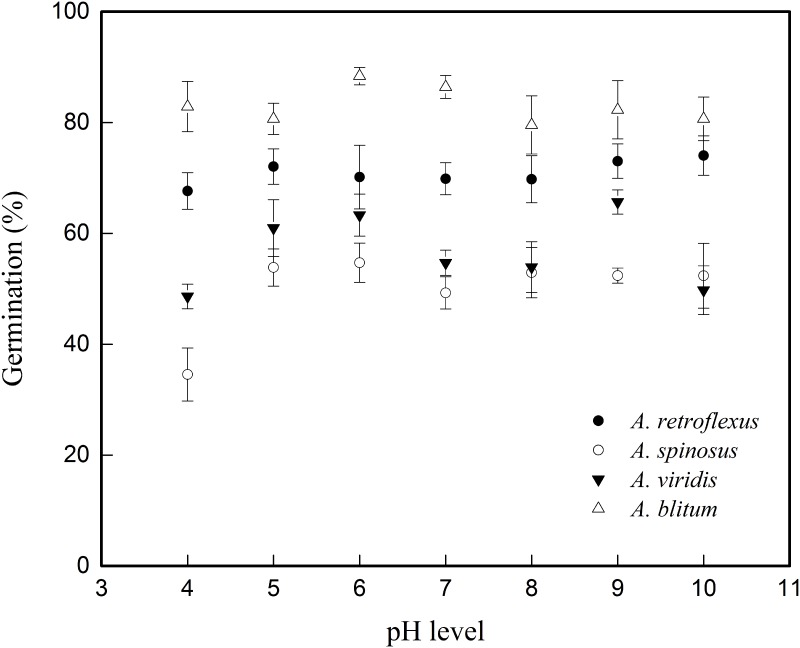
Figure showing three-parameter sigmoid model fitted data of Germination percentage (*G*_*max*_) in four *Amaranthus* species versus varying pH levels tested (pH4-pH10).

The results of seed germination at different pH treatments in amaranths showed that all four species can germinate at pH 4-pH10 solutions (Table 3 and Fig 2), demonstrating their broad adaptability to germinate in a wide range of soil pH conditions. The results of ANOVA analysis showed that there were significant differences in seed germination (*G*_*max*_) among treatments, from pH 4 to pH 10 levels (DF = 6, F = 2.890, p<0.05), and highly significant differences among four amaranth species (DF = 3, F = 74.070, p<0.01). However, there were no significant difference in seed germinations (*G*_*max*_) among pH×species (DF = 18, F = 1.136, p>0.05). *Amaranthus blitum* and *A*. *retroflexus* recorded best germination response than other two species. *A*. *spinosus* recorded the lowest germination response in all the pH ranges. The analysis of the results of multiple comparisons showed that there were no significant differences among 7 pH levels in both *A*. *retroflexus* (DF = 6, F = 0.346, p>0.05) and *A*. *blitum* (DF = 6, F = 0.733, p>0.05), but there were significant differences among pH levels in both *A*. *spinosus* (DF = 6, F = 3.438, p<0.05) and A. *viridis* (DF = 6, F = 3.131, p<0.05). The *A*. *retroflexus* and *A*. *blitum* had higher and quicker germination response than *A*. *spinosus* and *A*. *viridis* at pH 4–10 solution (Fig 2). Both *A*. *retroflexus* and *A*. *blitum* recorded highest *G*_*max*_ of 77.3% and 88.9%, respectively along with relatively lower *T*_*50*_ of 1.79–2.58 for *A*. *retroflexus* and between 1.78–1.90 for *A*. *blitum*. On the contrary, the highest *G*_*max*_ of *A*. *spinosus* and *A*. *viridis* were below 58.6% and 74.9% respectively with *T*_*50*_ ranging from 2.35–4.26 for *A*. *spinosus* and 4.9–26.77 for *A*. *viridis*. Except for *A*. *blitum*, the values of *T*_*50*_ of the other three amaranths were longer in pH4, pH 9, and pH10 than those in pH 5-pH8 (Table 3).

**Table 3:** Effect of pH on the germination of four *Amaranthus* species, incubated at 30/25°C in light/dark.

Species	Parameters	pH level						
		4	5	6	7	8	9	10
*A*. *retroflexus*	*G*_*max*_ (%)	69.90 (3.31)^**a,l**^	73.50 (3.20)^**a,kl**^	74.30 (5.75)^**a,kl**^	76.60 (2.89)^**a,l**^	77.30 (4.24)^**a,kl**^	76.80 (3.10)^**a,kl**^	76.00 (3.56)^**a,k**^
	*G*_*rate*_	0.35 (0.05)	0.13 (0.15)	0.13 (0.17)	0.33 (0.07)	0.12 (0.14)	0.42 (0.04)	0.041 (0.04)
	*T*_*50*_ (d)	1.97 (0.04)	1.79 (0.24)	1.81 (0.25)	1.90 (0.06)	1.79 (.024)	2.44 (0.05)	2.58 (0.05)
	*R*^*2*^	0.9939	0.9979	0.9967	0.9861	0.9982	0.9938	0.9934
*A*. *spinosus*	*G*_*max*_ (%)	34.55 (4.76)^**b,n**^	55.00 (3.36)^**a,l**^	58.60 (3.54)^**a,l**^	52.00 (2.90)^**ab,m**^	55.00 (4.52)^**a,l**^	55.10 (1.36)^**a,m**^	57.60 (5.84)^**a,l**^
	*G*_*rate*_	0.61 (0.04)	0.61 (0.07)	0.46 (0.06)	0.42 (0.05)	0.39 (0.03)	0.87 (0.10)	0.66 (0.06)
	*T*_*50*_ (d)	4.26 (0.05)	2.55 (0.08)	2.53 (0.07)	2.79 (0.06)	2.35 (0.04)	3.66 (0.12)	3.32 (0.07)
	*R*^*2*^	0.9972	0.9886	0.9886	0.9926	0.9967	0.9874	0.9930
*A*. *viridis*	*G*_*max*_ (%)	48.70 (2.20)^**b,m**^	66.60 (5.13)^**ab,l**^	70.50 (3.79)^**ab,l**^	64.80 (2.29)^**ab,m**^	59.80 (4.59)^**ab,l**^	74.90 (2.18)^**a,l**^	67.30 (4.40)^**ab, l**^
	*G*_*rate*_	0.81 (0.05)	1.10 (0.13)	0.72 (0.06)	0.61 (0.11)	0.98 (0.09)	1.49 (0.17)	1.41 (0.20)
	*T*_*50*_ (d)	5.64 (0.06)	5.10 (0.15)	4.92 (0.07)	5.05 (0.13)	5.39 (011)	6.77 (0.20)	6.52 (0.24)
	*R*^*2*^	0.9973	0.9876	0.9956	0.9839	0.9927	0.9864	0.9789
*A*. *blitum*	*G*_*max*_ (%)	84.00 (4.53)^**a,k**^	83.20 (2.82)^**a,k**^	88.90 (1.56)^**a,k**^	87.00 (2.07)^**a,k**^	80.40 (5.26)^**a,k**^	85.30 (5.25)^**a,k**^	80.70 (3.93)^**a, k**^
	*G*_*rate*_	0.25 (0.05)	0.12 (0.12)	0.09 (0.23)	0.09 (0.31)	0.12 (0.13)	0.20 (0.08)	0.19 (0.04)
	*T*_*50*_ (d)	1.90 (0.03)	1.80 (0.21)	1.80 (0.52)	1.78 (0.73)	1.80 (0.21)	1.84 (0.07)	1.88 (0.03)
	*R*^*2*^	0.9967	0.9992	0.9999	0.9994	0.9987	0.9938	0.9993

Table showing parameter estimates [*G*_*max*_, maximum germination (%); *G*_*rate*_, slope; *T*_*50*_, time to reach 50% of maximum germination (days)] of seed germination.

Values represent mean and standard error (parentheses).

Different lowercase letters (a-b) after the value of *G*_*max*_ indicated significant difference among treatments within same species (comparison within row).

Different lower case letters (k-n) after the value of *G*_*max*_ indicated significant differences among species within same treatment (comparison within column).

### NaCl treatment

**Fig 3:**
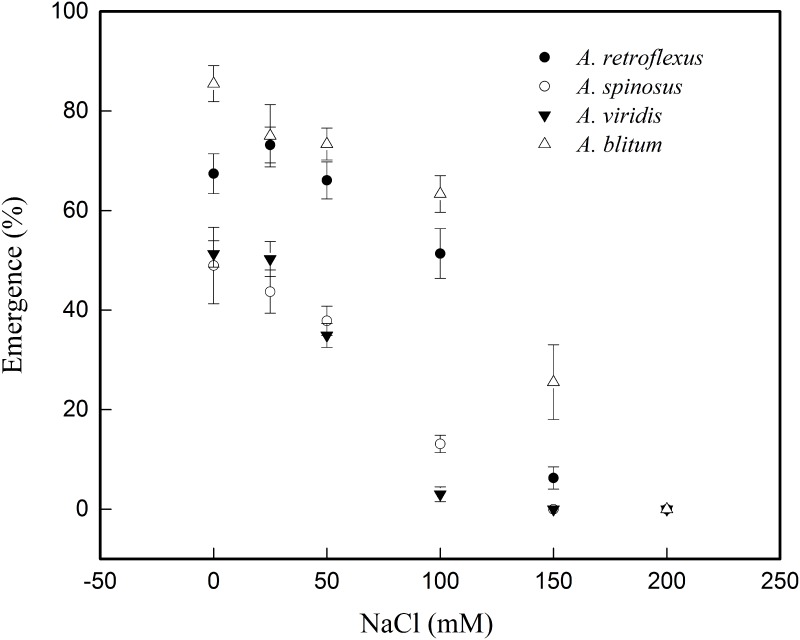
Figure showing effect of NaCl on germination response of four *Amaranthus* species fitted to three-parameter sigmoid model.

The results of seed germination at different NaCl treatments of four amaranths (Table 4 and Fig 3) showed that their *G*_*max*_ decreased and *T*_*50*_ increased with increasing NaCl concentrations, except in *A*. *retroflexus* and *A*. *blitum* that showed a little increase in *G*_*max*_ and at 150mM NaCl relative to other species. Although *A*. *retroflexus* recorded relatively lower *G*_*max*_ estimates than *A*. *blitum* at 150mM NaCl, it took less time to achieve 50% germination (*T*_*50*_). We observed highly significant difference in seed germinations (*G*_*max*_), both among different species (DF = 3, F = 110.403, p<0.01) and at different NaCl concentrations used (DF = 5, F = 242.173, p<0.01). There were highly significant difference in seed germinations (*G*_*max*_) among NaCl×species (DF = 15, F = 10.261, p<0.01). The NaCl concentrations which in which the *G*_*max*_ significantly decreased, with respect to control (0mM) were 100mM for *A*. *retroflexus*, 100mM for *A*. *spinosus*, 50mM for *A*. *viridis*, and 150mM for *A*. *blitum*, respectively. The *A*. *retroflexus* and *A*. *blitum* recorded higher and quicker germinations response than *A*. *spinosus* and *A*. *viridis* at all concentrations (Table 4).

**Table 4:** Effect of sodium chloride concentrations on the germination of four *Amaranthus* species, incubated at 30/25°C in light/dark.

Species	Parameters	NaCl Concentrations (mM)					
		0	25	50	100	150	200
*A*. *retroflexus*	*G*_*max*_ (%)	69.50 (4.00)^a,l^	74.70 (3.61)^a,k^	68.20 (3.72)^a,k^	59.00 (5.00)^b,l^	7.00 (2.24)^c,l^	0^c^
	*G*_*rate*_	0.26 (0.07)	0.15 (0.10)	0.30 (0.07)	1.08 (0.18)	1.54 (0.28)	0
	*T*_*50*_ (d)	1.86 (0.05)	1.81 (0.12)	1.87 (0.05)	3.59 (0.21)	6.21 (0.33)	0
	*R*^*2*^	0.9913	0.9967	0.9883	0.9682	0.9623	0
*A*. *spinosus*	*G*_*max*_ (%)	51.50 (7.67)^a,lm^	45.80 (4.35)^a,l^	38.00 (2.94)^a,l^	13.00 (1.74)^b,m^	0^b,l^	0^c^
	*G*_*rate*_	0.40 (0.05)	0.70 (0.05)	1.39 (0.19)	1.23 (0.14)	0	0
	*T*_*50*_ (d)	2.05 (0.05)	2.99 (0.06)	4.01 (0.21)	4.77 (0.16)	0	0
	*R*^*2*^	0.9931	0.9958	0.9748	0.9851	0	0
*A*. *viridis*	*G*_*max*_ (%)	63.70 (2.65)^a,m^	58.40 (3.52)^a,l^	47.10 (2.43)^b,l^	3.50 (1.49)^c,m^	0^c,l^	0^c^
	*G*_*rate*_	0.85 (0.05)	1.58 (0.22)	1.01 (0.13)	0.55 (0.07)	0	0
	*T*_*50*_ (d)	5.57 (0.06)	6.66 (0.26)	7.39 (0.15)	11.06 (0.08)	0	0
	*R*^*2*^	0.9976	0.9787	0.9884	0.9923	0	0
*A*. *blitum*	*G*_*max*_ (%)	87.30 (3.62)^a,k^	76.30 (6.23)^a,k^	74.30 (3.21)^a,k^	70.50 (3.68)^a,k^	25.90 (7.51)^b,k^	0^c^
	*G*_*rate*_	0.16 (0.10)	0.16 (0.07)	0.26 (0.03)	0.25 (0.07)	0.14 (0.39)	0
	*T*_*50*_ (d)	1.80 (0.12)	1.83 (0.08)	1.96 (0.05)	2.67 (0.11)	11.26 (0.72)	0
	*R*^*2*^	0.9957	0.9985	0.9987	0.9778	0.9674	0

Table showing parameter estimates [*G*_*max*_, maximum germination (%); *G*_*rate*_, slope; *T*_*50*_, time to reach 50% of maximum germination (days)] of seed germination.

Values represent mean and standard error (parentheses).

Different lowercase letters (a-c) after the value of *G*_*max*_ indicated significant differences among treatment within same species (comparison within row).

Different lower case letters (k-m) after the value of *G*_*max*_ indicated significant differences among species within same treatment (comparison within column).

### Osmotic treatment

**Fig 4:**
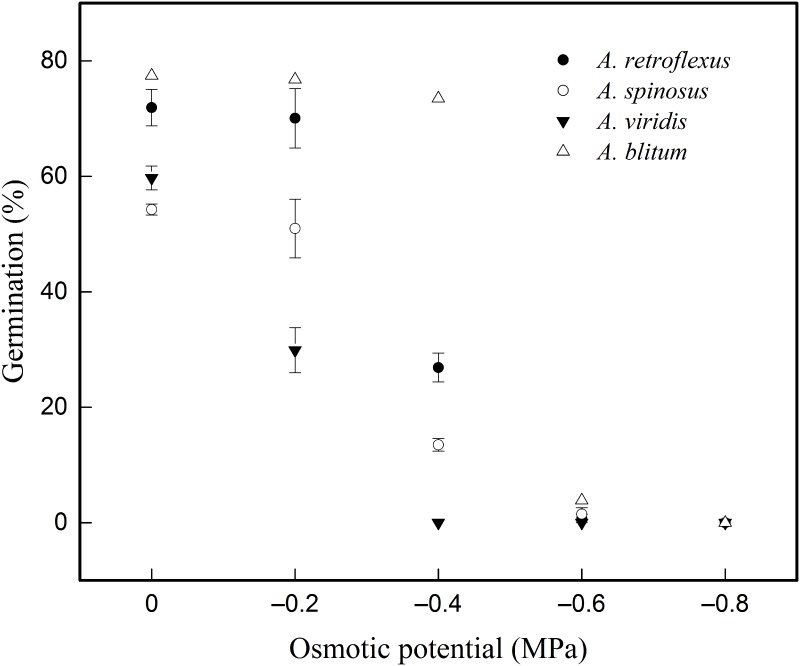
Effect of different Osmotic potential on germination response of *Amaranthus* species fitted to three-parameter sigmoid model.

The results of seed germination at different osmotic potential treatments in four amaranths showed that (Table 5 and Fig 4) in overall their *G*_*max*_ decreased and *T*_*50*_ increased with increasing osmotic potential. The analysis of main effects of general linear model showed that there were extremely significant difference in seed germinations (*G*_*max*_) in both among species (DF = 3, F = 103.972, p<0.01) and at different osmotic potentials (DF = 4, F = 482.920, p<0.01). Highly significant difference in seed germinations (*G*_*max*_) was recorded among osmotic potential×species (DF = 12, F = 26.118, p<0.01). The osmotic potentials at which the *G*_*max*_ significantly decreased compared to 0 potential were -0.4MPa for *A*. *retroflexus* and *A*. *spinosus*, -0.2MPa for *A*. *viridis*, and -0.6MPa for *A*. *blitum* respectively. No germination appeared at -0.4MPa for *A*. *viridis* while other three congeners having high or different degree of germination rate at the same potential, demonstrating lowest resistance to the moisture deficit for *A*. *viridis*. Noticeably *A*. *retroflexus* and *A*. *blitum* had similar higher and quicker germinations than *A*. *spinosus* and *A*. *viridis* at all potentials (Table 5).

**Table 5:** Effect of osmotic potential on the germination of four *Amaranthus* species incubated at 30/25°C in light/dark.

Species	Parameters	Osmotic potential (MPa)				
		0	-0.2	-0.4	- 0.6	-0.8
***A*. *retroflexus***	*G*_*max*_ (%)	72.30 (3.15)^a,k^	73.00 (5.13)^a,kl^	29.20 (2.50)^b,l^	0.00 (0.74)^c,lm^	0^c^
	*G*_*rate*_	0.14 (0.10)	0.43 (0.04)	0.58 (0.11)	0.55 (0.14)	0
	*T*_*50*_ (d)	1.81 (1.15)	2.30 (0.04)	3.63 (0.12)	7.97 (0.16)	0
	*R*^*2*^	0.9983	0.9956	0.9799	0.9759	0
***A*. *spinosus***	*G*_*max*_ (%)	56.30 (0.95)^a,l^	53.90 (5.08)^a,l^	13.50 (1.10)^b,m^	1.50 (1.10)^c,l^	0^c^
	*G*_*rate*_	0.47 (0.07)	0.70 (0.12)	0.56 (0.02)	0.04	0
	*T*_*50*_ (d)	2.15 (0.07)	2.81 (0.14)	3.98 (0.02)	6.97	0
	*R*^*2*^	0.9881	0.9740	0.9996	1.0000	0
***A*. *viridis***	*G*_*max*_ (%)	64.90 (2.06)^a,l^	41.20 (3.91)^b,m^	0^c,n^	0^c,l^	0^c^
	*G*_*rate*_	1.23 (0.10)	1.95 (0.25)	0	0	0
	*T*_*50*_ (d)	5.20 (0.12)	7.83 (0.33)	0	0	0
	*R*^*2*^	0.9923	0.9812	0	0	0
***A*. *blitum***	*G*_*max*_ (%)	79.30 (2.73)^a,k^	80.10 (1.78)^a,k^	74.70 (1.68)^a,k^	4.00 (0.82)^b,k^	0^b^
	*G*_*rate*_	0.14 (0.10)	0.28 (0.07)	0.31 (0.02)	0.24 (0.05)	0
	*T*_*50*_ (d)	1.80 (0.14)	1.87 (0.05)	3.79 (0.02)	3.46 (0.09)	0
	*R*^*2*^	0.9982	0.9888	0.9990	0.9852	0

Table showing parameter estimates [*G*_*max*_, maximum germination (%); *G*_*rate*_, slope; *T*_*50*_, time to reach 50% of maximum germination (days)] of seed germination.

Values represent mean and standard error (parentheses).

Different lowercase letters (a-c) after the value of *G*_*max*_ indicated significant differences among treatment within same species (comparison within row).

Different letters (k-n) after the value of *G*_*max*_ indicated significant differences among species within same treatment (comparison within column).

### Burial depth

**Fig 5:**
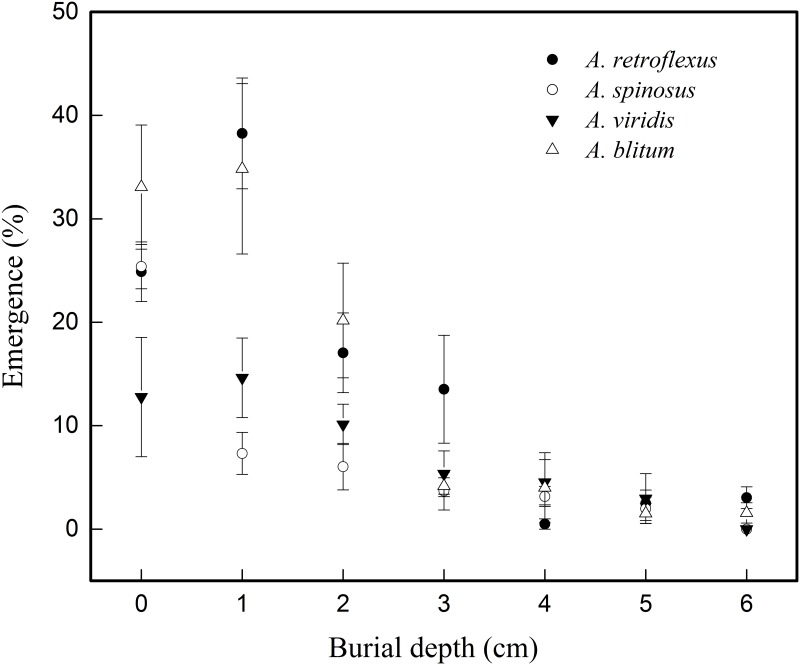
Figure showing effect of burial depth on germination response measured by seedling emergence (*E*_*max*_) in four *Amaranthus* species fitted to three-parameter sigmoid model.

The results of seed germination at different soil burial treatments of four amaranths (Table 6 and Fig 5) showed that the *E*_*max’s*_ increased at depth of 1 cm for *A*. *retroflexus*, *A*. *viridis*, and *A*. *blitum*. Their *E*_*max’s*_ decreased with the increase of burial depths when burial depths were more than 1 cm. On the contrary, highest *E*_*max*_ at soil surface recorded in *A*. *spinosus*, and that decreased with the increasing burial depths. The analysis of the result of general linear model showed that there were highly significant differences in seedling emergence (*E*_*max*_) among different species (DF = 3, F = 8.277, p<0.01) and also at different burial depths (DF = 6, F = 32.796, p<0.01). Our results also detected highly significant difference in seed seedling emergence (*E*_*max*_) among burial depths×species (DF = 18, F = 2.870, p<0.01). The burial depths in which the *E*_*max’s*_ significantly decreased compared to soil surface were 4 cm for *A*. *retroflexus*, 1cm for *A*. *spinosus*, 6 cm for *A*. *viridis*, and 3 cm for *A*. *blitum*, respectively (Table 6).

**Table 6:** Effect of burial depths on the seedling emergence of four *Amaranthus* species.

Species	Parameters	Burial depths (cm)						
		0	1	2	3	4	5	6
*A*. *retroflexus*	*E*_*max*_ (%)	26.00 (2.89)^**a,kl**^	39.00 (5.35)^a,k^	17.00 (3.86)^a,k^	13.50 (0.09)^ab,k^	0.50 (1.00)^b,k^	2.50 (1.29)^b,k^	3.04 (1.05)^b,k^
	*E*_*rate*_	1.67 (0.09)	1.39 (0.08)	1.90 (0.18)	1.05 (0.04)	0.02	0.78 (0.06)	1.77 (0.18)
	*T*_*50*_ (d)	17.72 (0.10)	17.31 (0.10)	17.92 (0.20)	17.97 (0.04)	17.50	18.22 (0.07)	15.20 (0.20)
	*E*_*min*_	-0.07 (0.19)	0.00 (0.30)	-0.16 (0.24)	-0.05 (0.05)	-4.25	-0.05 (0.02)	-0.04 (0.05)
	*R*^*2*^	0.9968	0.9961	0.9898	0.9991	1.0000	0.9967	0.9886
*A*. *spinosus*	*E*_*max*_ (%)	27.50 (2.21)^a,kl^	7.00 (2.03)^b,l^	6.04 (2.25)^bc,k^	3.50 (1.84)^bc,k^	3.00 (0.97)^bc,k^	2.00 (1.13)^bc,k^	0^c,k^
	*E*_*rate*_	1.66 (0.14)	3.43 (0.38)	1.84 (0.15)	4.94 (1.06)	4.39 (0.84)	0.77 (0.11)	0
	*T*_*50*_ (d)	18.66 (0.16)	17.63 (0.39)	18.28 (0.17)	14.66 (0.88)	14.97 (0.75)	13.66 (0.13)	0
	*E*_*min*_	0.20 (0.28)	0.07 (0.16)	0.16 (0.07)	-0.33 (0.28)	-0.15 (0.19)	-0.02 (0.03)	0
	*R*^*2*^	0.9924	0.9852	0.9924	0.9609	0.9634	0.9882	0
*A*. *viridis*	*E*_*max*_ (%)	13.00 (5.76)^a,l^	15.00 (3.84)^a,l^	10.50 (1.95)^ab,k^	5.50 (2.21)^ab,k^	4.53 (2.19)^ab,k^	3.00 (2.41)^ab,k^	0^b,k^
	*E*_*rate*_	2.83 (0.58)	3.06 (0.26)	2.52 (0.28)	1.00 (0.07)	2.65 (0.30)	0.34 (0.07)	0
	*T*_*50*_ (d)	17.25 (0.05)	22.02 (0.34)	23.87 (0.40)	22.39 (0.04)	21.35 (0.37)	20.51 (0.10)	0
	*E*_*min*_	-1.07 (0.40)	-0.23 (0.17)	0.05 (0.12)	-0.20 (0.04)	-0.07 (0.07)	0.07 (0.04)	0
	*R*^*2*^	0.9421	0.9915	0.9858	0.9955	0.9841	0.9850	0
*A*. *blitum*	*E*_*max*_ (%)	33.50 (6.00)^a,k^	35.50 (8.24)^a,k^	20.00 (5.54)^ab,k^	4.00 (0.80)^b,k^	4.00 (2.19)^b,k^	1.50 (0.97)^b,k^	1.50 (0.98)^b,k^
	*E*_*Grate*_	1.51 (0.07)	1.09 (0.04)	1.57 (0.05)	2.08 (0.23)	1.22 (0.08)	1.14 (0.15)	1.93 (0.41)
	*T*_*50*_ (d)	17.67 (0.08)	17.20 (0.05)	18.01 (0.05)	20.33 (0.26)	19.10 (0.09)	19.06 (0.18)	19.14 (0.46)
	*E*_*min*_	-0.19 (0.21)	0.02 (0.16)	-0.09 (0.08)	0.03 (0.06)	-0.05 (0.03)	0.00 (0.02)	0.05 (0.05)
	*R*^*2*^	0.9977	0.9988	0.9989	0.9854	0.9965	0.9851	0.9493

Table showing parameter estimates [*E*_*max*_, the difference of maximum and minimum emergence (%); *E*_*rate*_, slope; *T*_*50*_, time (d) required for 50% of maximum seedling emergence, *E*_*min*_, minimum seedling emergence (%)] of seedling emergence.

Values represent mean and standard error (parentheses).

Different lowercase letters (a-c) after the value of *E*_*max*_ indicated significant differences among treatments within same species (comparison within row).

Different letters (k-l) after the value of *E*_*max*_ indicated significant differences among species within same treatment (comparison within column).

## Discussion

Seed germination is an integrated process influenced by biotic and abiotic factors as well as dependent on the genetic and physiological state of readiness [42, 43]. Germination characteristics affect plant propagation and distribution especially for annual species reproducing exclusively by seed [44]. This study demonstrated the effects of some environmental factors on the germination response in four wild *Amaranthus* species. Our experimental findings provide evidence that *Amaranthus* species have variable potential to grow in a variety of environmental regime. The results showed that the *A*. *blitum* and *A*. *retroflexus* have better overall germination performances (higher *G*_*max*_, rapider *G*_*rate*_ and shorter *T*_*50*_) compare to *A*. *spinosus* and *A*. *viridis* in various treatments. Low invasive weed *A*. *blitum* recorded the best overall germination performance, with the highest *G*_*max*_, the rapidest *G*_*rate*_, and the shortest *T*_*50*_. Highly invasive *A*. *retroflexus* did not differ significantly from *A*. *blitum* in most treatments, except for pH (pH4-pH7), NaCl (0mM, 100mM, 150mM) and osmotic potential (-0.4 and -0.6MPa). The overall germination performances of *A*. *spinosus* and *A*. *viridis* were similar and consistently low across all treatments. In most treatments, the *G*_*max*_ of *A*. *spinosus* and *A*. *viridis* were not extremely different except for photoperiod 16/8, pH 4, pH 9, osmotic potential -0.2 and -0.4MPa.

The germination results of the present study were mostly in accordance with the previous studies in *A*. *retroflexus* [26], *A*. *spinosus* [32], and *A*. *viridis* [31], [32]. In a study by Ghorbani et al. [26], the germination of *A*. *retroflexus* found to decrease with increasing moisture deficit, and the germination rates (*G*_*max*_) at 25°C were 68%, 52%, 50%, 33%, 14%, 8%, and 0% at 0MPa, -0.1MPa, -0.2MPa, -0.3MPa, -0.4MPa, -0.5MPa, and -1MPa osmotic potential respectively. The seedlings of *A*. *retroflexus* emerged only at depths of lower than 5cm. In the study of Chauhan and Johnson [32], germination of *A*. *viridis* was more sensitive to increasing salt and water stress than *A*. *spinosus*. Our present results confirmed their observation. The *G*_*max*_ of *A*. *viridis* declined sharply from 47.1% at 50mM to 3.5% at 100mM NaCl concentration, whereas *A*. *spinosus* declined slowly from 38.0% at 50mM to 13.0% at 100mM NaCl. Meanwhile, the *G*_*max*_ of *A*. *spinosus* was 13.5% at -0.4MPa osmotic potential, whereas seeds of *A*. *viridis* did not germinate at all at this potential. Similar germination response of *A*. *viridis* to water stress was also demonstrated in the study of Thomas et al. [31]. In their study, extremely low germination (1.25% at 30/20°C) at -0.4MPa was reported which was the lethal osmotic potential causing complete inhibition in our study. The only difference observed is in the germination response of *A*. *viridis* to different pH values between our study and that of Thomas et al. [31]. In their study, the average values of *G*_*max*_ from pH3 to pH9 were 68.8%, 63.8%, 79.5%, 58.1%, 49.0%, 39.6%, and 52.8%, respectively. They concluded that the germination was greater with acidic than with basic pH. The average values of *G*_*max*_ from pH 4 to pH 10 in our study were recorded as 48.7%, 66.6%, 70.5%, 64.8%, 59.8%, 74.9%, and 67.3% in *A*. *viridis*, respectively. Our results showed that there were no significant differences among different pH values, except between pH 4 with pH 9 suggesting they are adapted to both acidic and basic pH ranges, but with lower germination response at pH 4 and relatively higher at pH 9. The results of burial depth were also in conformity to Chauhan and Johnson’s [32], and showed that emergence of *A*. *spinosus* was affected to greater extent by increasing seed burial depths. The germination of *A*. *spinosus* was the highest at soil surface (27.5% in our study and 56.0% in Chauhan and Johnson study). The emergence rate significantly declined when seeds were covered by soil, even if at 0.5 cm depth (7.0% in ours as well as in Chauhan and Johnson’s study). However, there were no significant differences for germination at depths from 0 cm to 3 cm for *A*. *retroflexus*, at depths from 0 cm to 5 cm for *A*. *viridis* and at depths from 0 cm to 2 cm for *A*. *blitum*. The reason for this likely related to the seed size. Seed size was associated with germination traits and seedling growth in non-competitive cover [45]. Lower seedling emergence of seeds at deep depths may be linked to limited seed reserves [45, 46]. Larger seeds often have greater reserves and are able to emerge from greater depths [46]. The seeds of *A*. *spinosus* were significantly smaller and relatively lighter (129mg for 1000-seed weights) than those of the other three amaranths (312mg for *A*. *retroflexus*, 388mg for *A*. *viridis*, and 378g for *A*. *blitum* respectively) and that might be a reason for *A*. *spinosus* which had highest emergence rate on the soil surface and emergence decreased with increasing burial depth.

According to Pyšek and Richardson [47] germination of alien invasive species was more rapid, higher and successful across more environmental conditions than that of congeneric native/noninvasive taxa. However, in our present study, it appeared that invasiveness of amaranth was not always positively correlated, or at least partly, with their germination performance in wide environmental conditions as demonstrated in two amaranth species (*A*. *retroflexus* and *A*. *blitum*) with contrasting invasiveness but exhibiting equivalently high germination performance. However, when considering the global records and occurrences of these four amaranths, it showed that these two species with higher germination performances (*A*. *retroflexus* and *A*. *blitum)* have more respective global records (31183 & 13557) and occurrences (36070 & 16639) than the other two congeners (*A*. *spinosus* and *A*. *viridis*) with lower germination performances (Table 1). This implied that high germination potential and adaptability to a wide soil condition might play a key role contributing to population establishment and colonization and one of the reasons for widespread distribution of *A*. *retroflexus* and *A*. *blitum*.

Rapid distribution and invasion by weeds and invasive plants is an increasingly serious problem and has attracted considerable attention worldwide [48]. Physiochemical properties of soil influenced by growing microbial communities and other edaphic factors cause change in pH, salinity, and nutrient level that have profound effect on seedling growth and emergence of weedy/invasive species [49]. Besides germination performance, other reproductive traits such as seed production, seed viability, and dynamics of soil seed bank were important ones which may influence the distribution and invasion success in the new region [17, 4, 5]. In our common garden experiment on four amaranth species, the invasiveness (Table 1) was positively correlated with the seed production (78,063±18,013 for *A*. *retroflexus*, 22,777±9,451 for *A*. *spinosus*, and 9152± 4407 for *A*. *viridis*). However, the seed production of *A*. *blitum* (5357± 2104) was relatively less and only found to be 6.9% of *A*. *retroflexus*, 23.5% of *A*. *spinosus* and 58.5% of *A*. *viridis*. Therefore, the seed germination potential, maybe along with high seed production, plays the union role in wide distribution and invasion success of highly invasive *A*. *retroflexus*.

Our experimental results confirmed that the superior germination potential under a wide spectrum of environmental conditions is likely to make it difficult to control *A*. *retroflexus*, in agricultural field and in non-native habitat. Practices like deep burial of seeds by turning over the top soil and using of tillage agriculture system are some of the potential options to inhibit the emergence and growth of these weed species.

## Conclusion

According to our congeneric comparative results in four amaranths, the germination performances among different species differing in invasiveness didn’t show complete positive correlation with the invasiveness. Therefore, the germination performances can’t be used directly or solely as an indicator of invasiveness, but could be considered as a reference indicator. An integrated consideration of the role of germination and emergence response to various environmental factors combined with other factors is necessary to assess the weediness and invasive characteristics of weedy/invasive plants. In addition, it shall be noted that large scale multi-species empirical comparative experiments involving invasive, non-invasive and native species was imperative for the understanding of invasion mechanism and risk assessment of plant invasion.
